# Eruptions Due to Antipyrin

**Published:** 1909-08-21

**Authors:** 


### DERMATOLOGY.

### ERUPTIONS DUE TO ANTIPYRIN.

Antipyrin is a drug which is often taken by patients for minor ailments without advice from a medical man, and it is well to bear in mind that it is capable of giving rise in some persons to various forms of skin eruption. There are three principal types of eruption produced by the ingestion of antipyrin. One is a scarlatiniform erythema, accompanied by burning and itching, often involving the mucous membrane of the lips, mouth, and throat, frequently with some fever, and followed by desquamation. In other cases there may be a morbilliform rash, consisting of dusky red, irregularly rounded blotches, which may be almost generalised, or may be situated mainly on the limbs. This eruption may appear in the same patient every time the drug is taken. It comes out quickly and disappears slowly. A third form of eruption has been called " fixed erythema," and it may consist of a single patch, or at most a small number. The eruption appears from half-an-hour to a few hours after the drug has been taken. The patches are rounded or oval, red, infiltrated, raised, and sometimes even bullous. The lesion fades slowly in the course of several days or a week or more, leaving behind a pigmented macule, which may remain for many months. A striking feature of this form of " fixed erythema " is that at every fresh attack the patches appear exactly upon the same area, although in the course of time, if the drug be continued, fresh patches may appear. After several attacks the pigmentation becomes altogether indelible. In some few instances gangrene of the patch has occurred. In the presence of an eruption of rounded, infiltrated patches, of sudden origin, and with tendency to recur in the same spots, one should think, therefore, of antipyrin.

